# *In vitro* Exposure to Inflammatory Mediators Affects the Differentiation of Mesenchymal Progenitors

**DOI:** 10.3389/fbioe.2022.908507

**Published:** 2022-06-22

**Authors:** S. Marsh, T. Constantin-Teodosiu, V. Chapman, V. Sottile

**Affiliations:** ^1^ School of Medicine, University of Nottingham, Nottingham, United Kingdom; ^2^ Pain Centre Versus Arthritis, University of Nottingham, Nottingham, United Kingdom; ^3^ School of Life Sciences, University of Nottingham, Nottingham, United Kingdom; ^4^ Department of Molecular Medicine, University of Pavia, Pavia, Italy

**Keywords:** differentiation, mesenchymal progenitors, interleukins, inflammation, osteoarthritis, *in vitro* model

## Abstract

The increasing prevalence of joint disease, and in particular osteoarthritis (OA), calls for novel treatment strategies to prevent disease progression in addition to existing approaches focusing mainly on the relief of pain symptoms. The inherent properties of mesenchymal stem cells (MSCs) make them an attractive candidate for novel tissue repair strategies, as these progenitors have the potential to differentiate into chondrocytes needed to replace degraded cartilage and can exert a modulating effect on the inflammatory environment of the diseased joint. However, the inflammatory environment of the joint may affect the ability of these cells to functionally integrate into the host tissue and exert beneficial effects, as hinted by a lack of success seen in clinical trials. Identification of factors and cell signalling pathways that influence MSC function is therefore critical for ensuring their success in the clinic, and here the effects of inflammatory mediators on bone marrow-derived MSCs were evaluated. Human MSCs were cultured in the presence of inflammatory mediators typically associated with OA pathology (IL-1β, IL-8, IL-10). While exposure to these factors did not produce marked effects on MSC proliferation, changes were observed when the mediators were added under differentiating conditions. Results collected over 21 days showed that exposure to IL-1β significantly affected the differentiation response of these cells exposed to chondrogenic and osteogenic conditions, with gene expression analysis indicating changes in MAPK, Wnt and TLR signalling pathways, alongside an increased expression of pro-inflammatory cytokines and cartilage degrading enzymes. These results highlight the value of MSCs as a preclinical model to study OA and provide a basis to define the impact of factors driving OA pathology on the therapeutic potential of MSCs for novel OA treatments.

## Introduction

Although osteoarthritis is the most prevalent form of joint disease, the molecular mechanisms underpinning OA pathogenesis are still elusive (Chen et al., 2017; He et al., 2020). Consequently, strategies to prevent disease progression and preserve cartilage are lacking, with treatment mainly relying on the pain relief provided by pharmacological or surgical interventions (Charlesworth et al., 2019; Grässel and Muschter, 2020; National Institute for Health and Care Excellence, 2020). Given that endogenous cartilage regeneration is limited (Heng et al., 2004; Kock et al., 2012), novel approaches aiming to restore joint tissue through the use of mesenchymal stem cells (MSCs) are emerging. These progenitors can differentiate into osteoblasts and chondrocytes, and therefore have the potential to promote the regeneration of damaged cartilage (Hwang et al., 2021; Zhu et al., 2021; Xiang et al., 2022). Pre-clinical studies have revealed that while endogenous populations of synovial MSCs can contribute to cartilage repair following chondrogenesis (Harrell et al., 2019), the exogenous delivery of MSCs may result in a reduction in cartilage degradation and pain (Chapman et al., 2017; Harrell et al., 2019; Wang et al., 2022). OA also has a strong inflammatory component (Sokolove and Lepus, 2013), with synovial infiltration of immune cells such as macrophages, T cells and B cells (De Lange-Brokaar et al., 2012; Zhao et al., 2020). MSCs have been shown to act on cells of both the innate and adaptive immune systems and promote an anti-inflammatory environment, through cell-cell contact as well as paracrine mechanisms (Gao et al., 2016; Wang et al., 2018; Song et al., 2020; Zhao et al., 2020). Therefore, MSCs may present an important therapeutic potential as they could promote a regenerative microenvironment primed for cartilage repair; however, retention of these cells in an osteoarthritic joint *in vivo* remains uncertain (Mancuso et al., 2019).

As such, MSCs have been the focus of many clinical trials assessing their efficacy and safety in humans. While these trials appear to support the safety of MSC-based therapies and indicate some modulation of OA through a reduction in pain, it is not yet possible to ascertain the efficacy of MSC treatment in OA due to a current lack of reproducibility and long-term follow-up, as well as high-risk bias (Iijima et al., 2018). Recent meta-analyses have exposed a high variability between trial methods, including MSC source, preparation, cell density, and implantation techniques (Iijima et al., 2018; Wang et al., 2020; Zhu et al., 2021). Current studies have also been unable to demonstrate a positive impact of MSC treatment on cartilage repair (Gupta et al., 2016; Matas et al., 2019; Wang et al., 2020), which may be partly linked to the OA environment in which these cells are introduced.

Activation of the immune response in the OA joint results in the production of destructive inflammatory mediators and proteases (Sokolove and Lepus, 2013). This is, in part, regulated by the expression of toll-like receptors (Barreto et al., 2020), which are also important modulators of MSC differentiation and immunoregulation (DelaRosa et al., 2012; Sangiorgi and Panepucci, 2016; Shirjang et al., 2017). Upregulated inflammatory mediators include the pro-inflammatory cytokine IL-1β, which is markedly increased in the chondrocytes and synovium of OA patients (Kapoor et al., 2011; Wojdasiewicz et al., 2014). IL-1β levels have been shown to have a positive correlation with OA changes (Daheshia and Yao, 2008; Denoble et al., 2011). Similarly, the pro-inflammatory chemokine IL-8 (or CXCL8) is high in the synovium of OA patients (Kaneko et al., 2000; Pierzchala et al., 2011; Molnar et al., 2021) and its level is linked to clinical severity (García-Manrique et al., 2021). IL-10 is also present in the osteoarthritic joint, but is considered an anti-inflammatory cytokine (Iannone et al., 2001) known to inhibit inflammatory arthritis progression in mice (Choi et al., 2008). This cytokine was also shown to stimulate the production of proteoglycans in articular cartilage (Van Roon et al., 1996), suggesting that it could exert an overall mitigating effect in OA by improving both cartilage and immunological parameters. Since the OA joint presents an altered level of inflammatory mediators, the present study aimed to investigate the effects of leading interleukins IL-1β, IL-8 and IL-10 on MSCs *in vitro*, focusing on their impact on cell differentiation and the possible underlying mechanisms of action.

## Methods

Reagents were purchased from Thermo Fisher Scientific (Hemel Hempstead, United Kingdom) unless otherwise stated.

### Cell Culture

Immortalised human bone marrow MSCs (Macri-Pellizzeri et al., 2018) and human primary bone marrow-derived MSCs (BMSCs, Lonza, United Kingdom) were cultured in standard medium (SM) consisting of low glucose (1 g/L) Dulbecco’s Modified Eagle Medium (DMEM) supplemented with 10% (v/v) fetal bovine serum, 1% (v/v) non-essential amino acids, 1% (v/v) L-glutamine and 1% (v/v) penicillin-streptomycin. Cells were passaged using 0.25% (v/v) trypsin/0.02% (v/v) EDTA. Recombinant human IL-1β (Abnova), recombinant human IL-8 (R&D systems) or recombinant human IL-10 (Source Bioscience) were reconstituted according to manufacturer’s guidelines and added to the medium at dose ranges based on previous studies (De Jager et al., 2003; Meijer et al., 2003; Rutgers et al., 2010) as indicated in the different experiments. Cells were kept at 37.5°C, 5% CO_2_, and the medium was changed every 2–3 days.

### Cell Proliferation and Viability

Cells were seeded into well plates and subsequently counted and passaged every 48 h. Cell proliferation was evaluated using the Countess II FL automated system. Cell metabolic activity was analysed using the PrestoBlue Cell Viability reagent, according to the manufacturer’s instructions.

### Osteogenic Differentiation

Cells were seeded at a density of 4,000 cells/cm^2^ onto tissue culture plastic. After 24 h, SM was removed and osteogenic medium (OM), with or without recombinant IL-1β, IL-8 or IL-10, was added to induce osteoblast differentiation. Osteogenic medium consisted of SM supplemented with 100 nM dexamethasone (Sigma-Aldrich), 0.05 mM l-ascorbic acid-2-phosphate (Sigma-Aldrich) and 10 mM β-glycerophosphate (Sigma-Aldrich). The medium was changed every 2–3 days for 21 days.

### Alkaline Phosphatase Assay and Alizarin Red S Staining

Early osteogenic differentiation was assessed on days 10 and 14 by alkaline phosphatase activity and measured using p-nitrophenyl phosphate tablets (Sigma-Aldrich), according to manufacturer’s instructions. Mineral deposition was assessed on days 10, 14 and 21 as previously described (Macri-Pellizzeri et al., 2018), using 1% Alizarin Red solution and imaged using an Eclipse TS100 inverted microscope (Nikon). Alizarin Red signal was quantified using an Infinite 200 microplate reader at 405 nm.

### Chondrogenic Differentiation

Cells were pelleted in 1.5 ml Eppendorf tubes at 1 × 10^6^ cells/tube. After 24 h, SM was removed and chondrogenic medium (CM), with or without recombinant IL-1β, IL-8 or IL-10, was added to induce chondrocyte differentiation. Chondrogenic medium consisted of high glucose (4.5 g/L) DMEM supplemented with 1% (v/v) non-essential amino acids, 1% (v/v) L-glutamine, 1% (v/v) penicillin-streptomycin, 110 mg/L (w/v) sodium pyruvate, 0.1 μM dexamethasone, 50 μM l-ascorbic acid-2-phosphate, 40 μg/ml (w/v) L-proline, 1% (v/v) ITS supplement and 10 ng/ml TGF-β1 (Prosser et al., 2019). The medium was changed three times a week for 21 days.

### DMMB Assay and PicoGreen Assay

Chondrogenic differentiation was assessed through the measurement of sulfated glycosaminoglycans (sGAGs) extracted using a papain extraction reagent made of 0.2 M sodium phosphate buffer with 8 mg/ml (w/v) sodium acetate (Sigma-Aldrich), 4 mg/ml (w/v) EDTA (Acros Organics), 0.8 mg/ml (w/v) cysteine hydrochloride (Sigma-Aldrich) and 100 μg/ml papain (Sigma-Aldrich), at pH 6.4. Pellets were incubated in 1 ml papain extraction reagent at 65°C for 18 h, then centrifuged at 10,000 × *g* for 10 min. The supernatant was decanted for use with the Blyscan Sulfated Glycosaminoglycan Assay kit (Biocolor Life Science Assays, United Kingdom) according to the manufacturer’s instructions. sGAG concentration was normalised to the nucleic acid concentration, measured with the QuantiT PicoGreen^®^ dsDNA kit according to the manufacturer’s instructions.

### TaqMan Low Density Arrays

MSCs were washed twice with PBS and suspended in Tri-Reagent for subsequent RNA extraction using the RNeasy Micro Kit (Qiagen) according to the manufacturer’s instructions. RNA concentration and purity were assessed using a Nanodrop ND-1000 spectrophotometer before performing cDNA synthesis using a High Capacity RNA-to-cDNA Kit, according to manufacturer’s instructions. Custom TaqMan Low Density Array (TLDA) 384-well microfluidic cards with a 96 gene × 4 sample layout from Thermo Fisher Scientific were used for the analysis of MSC gene expression. Where possible, probes spanning an exon boundary and outside of the 5′ untranslated region were chosen. The cards were loaded with 200 ng of cDNA diluted in 50 μl RNase free water and 50 μl of TaqMan Universal Master Mix II with uracil-N-glycosylase in each reservoir. cDNA was dispersed across the wells by centrifugation at 200 × g for two 1 min spins, after which the plate was sealed and run in a 7900HT Fast Real-Time PCR System using SDS RQ Manager Software (Applied Biosystems). Gene expression was calculated as fold change from untreated cells (Pfaffl, 2001), normalised to the geometric mean of the two most stable reference genes (Vandesompele et al., 2002).

### Statistical Analysis

All data are presented as mean ± SEM. Statistical analysis was performed using GraphPad Prism Software (https://www.graphpad.com), using a two-tailed Student’s *t*-test with significance as *p < 0.05; **p < 0.01; ***p < 0.001; ****p < 0.0001.

## Results

### MSC Response to Interleukin Treatment *in vitro*

**FIGURE 1:**
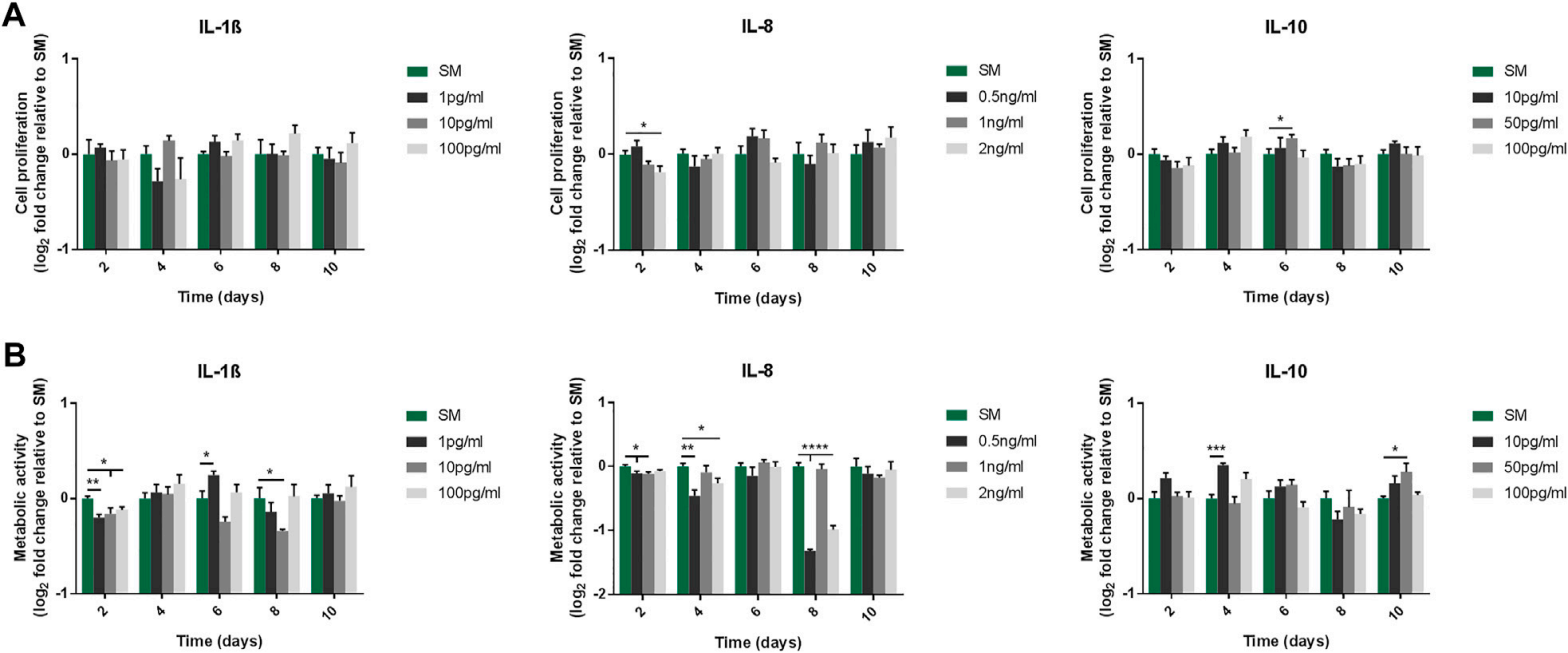
Proliferative capacity **(A)** and metabolic activity **(B)** of MSC cultures following exposure to IL-1β, IL-8 and IL-10 for 10 days. **(A)** Proliferative capacity measured by cell counts. Doses used were 1/10/100 pg/ml for IL-1β, 0.5/1/2 ng/ml for IL-8, and 10/50/100 pg/ml for IL-10. Data shown as log_2_ fold change compared to SM control condition. Error bars represent SEM. *n* = 4, *p < 0.05, **p < 0.01, ***p < 0.001, ****p < 0.0001.

To evaluate the effect of IL-1β, IL-8 and IL-10 on MSCs, cells were cultured with increasing doses of each interleukin, and their proliferative capacity and metabolic activity were assessed over 10 days. Both sets of measurements indicated that the proliferative capacity (Figure 1A) and metabolic activity (Figure 1B) of cells exposed to increasing doses of any of the mediators remained overall unchanged compared to cells cultured in SM.

### Effects of Interleukin Supplementation on Mesenchymal Stem Cell Chondrogenic Differentiation

**FIGURE 2:**
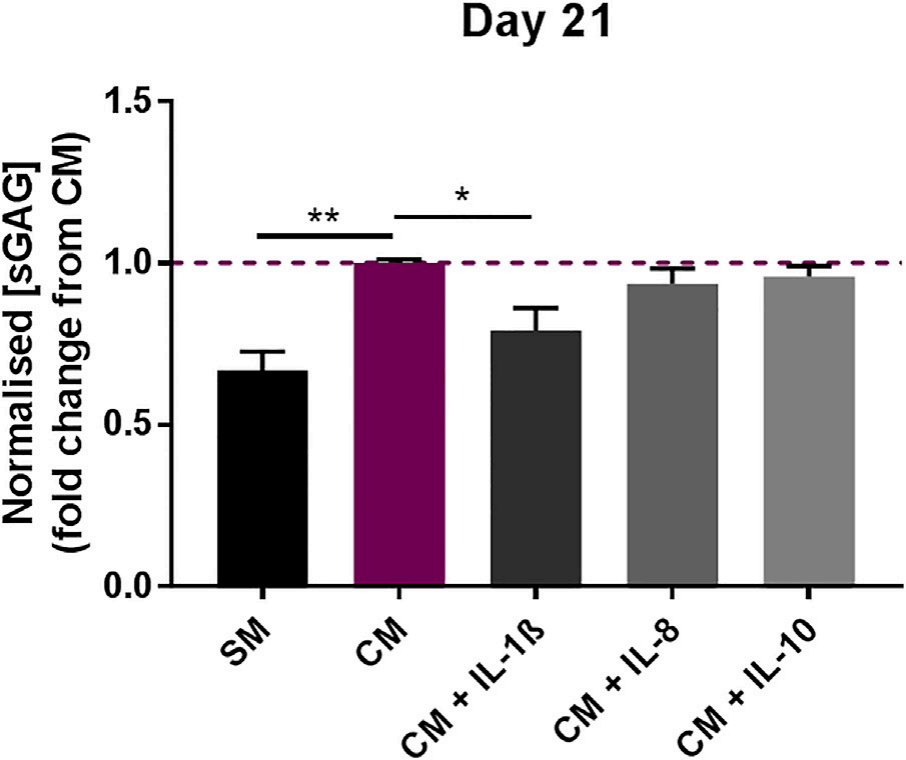
Effects of IL-1β (100 pg/ml), IL-8 (2 ng/ml) and IL-10 (100 pg/ml) supplementation on MSC chondrogenic response. sGAG concentration in MSC pellet cultures in the presence or absence of IL-1β, IL-8 and IL-10 measured after 21 days, normalised to DNA content. Data shown as fold change compared to CM control condition. Error bars represent SEM. n = 4, *p < 0.05, **p < 0.01.

The effect of the interleukins on MSC chondrogenic differentiation was analysed by treating pellet cultures maintained under pro-chondrogenic conditions, and measuring the level of sulfated glycosaminoglycans (sGAGs) after 21 days of differentiation. The concentration of sGAGs was normalised to DNA content to account for possible variation in cell numbers in the different samples analysed. Whilst the addition of IL-8 (2 ng/ml) and IL-10 (100 pg/ml) did not significantly alter the chondrogenic response compared to CM control, exposure to IL-1β (100 pg/ml) significantly decreased the chondrogenic response, as indicated by the decrease in sGAGs measured at day 21 (0.79 ± 0.07 fold change compared to CM) (Figure 2).

### Effects of Interleukin Supplementation on Mesenchymal Stem Cell Osteogenic Differentiation

**FIGURE 3:**
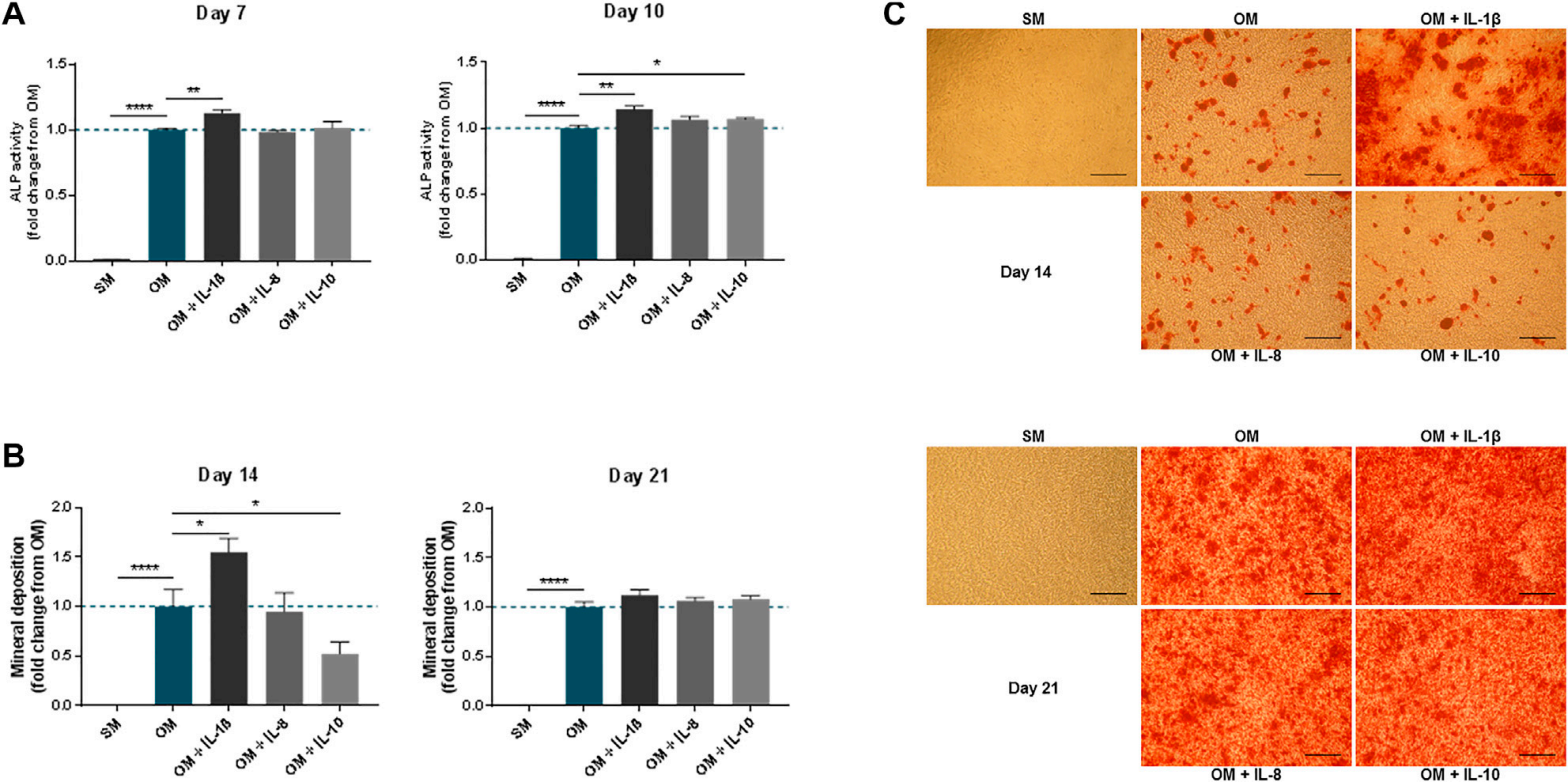
Effects of IL-1β, IL-8 or IL-10 supplementation on MSC osteogenic medium. ALP activity **(A)** and mineral deposition **(B)** measured in MSC cultures in the presence or absence of IL-1β (100 pg/ml), IL-8 (2 ng/ml) or IL-10 (100 pg/ml) measured over up to 3 weeks. **(C)** Representative images of Alizarin Red staining at days 14 and 21. Scale bar = 150 μm. Data shown as fold change compared to the OM control condition. Error bars represent SEM. *n* ≥ 4, *p < 0.05, **p < 0.01, ***p < 0.001, ****p < 0.0001.

To analyse the impact of the interleukins on the MSC osteogenic response, the levels of alkaline phosphatase activity (ALP), used as early marker, and mineral deposition, used as late marker, were measured in osteogenic cultures supplemented with each molecule. The addition of IL-1β (100 pg/ml) significantly increased ALP activity in MSCs compared to OM alone at both time-points analysed (1.13 ± 0.02 fold change compared to OM at day 7 and 1.14 ± 0.02 fold change compared to OM at day 10) (Figure 3A). This was mirrored by an increase in mineralisation measured at day 14 (1.55 ± 0.14 fold change compared to OM) and then lost by day 21 (Figures 3B,C). By contrast, supplementation with IL-8 (2 ng/ml) did not significantly alter the levels of ALP activity or mineral deposition in MSCs compared to OM controls. IL-10 (100 pg/ml) supplementation did show a modest increase in ALP activity by day 10 only (1.07 ± 0.01 fold change compared to OM), while mineral deposition showed a transient decrease at day 14 (0.52 ± 0.12 fold change compared to OM), which recovered by day 21.

### Effects of IL-1β Supplementation on Primary Cultures

**FIGURE 4:**
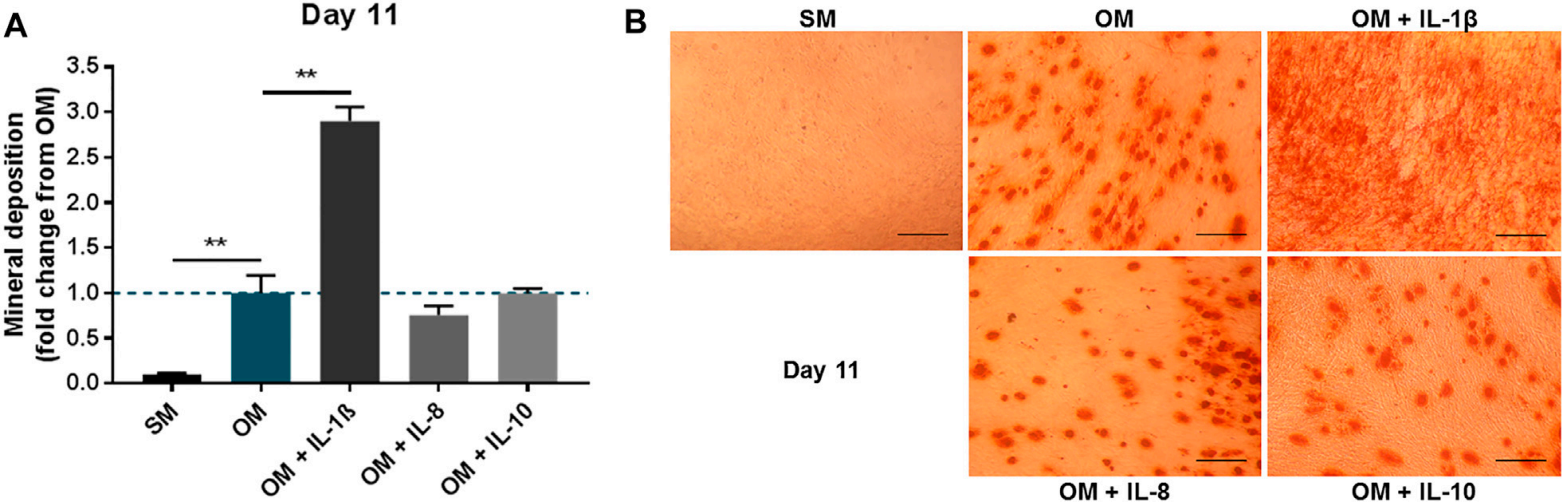
Effects of IL-1β, IL-8 or IL-10 supplementation on primary MSC cultures treated in osteogenic medium. **(A)** Mineral deposition in cultures treated in the presence or absence of IL-1β (100 pg/ml), IL-8 (2 ng/ml) or IL-10 (100 pg/ml) measured after 11 days. **(B)** Representative images of Alizarin Red staining at day 11. Scale bar = 150 μm. Data shown as fold change compared to the OM control condition. Error bars represent SEM. *n* = 3, **p < 0.01.

To further verify the effect of the interleukins on MSCs, inflammatory mediators (IL-1β, IL-8, IL-10) were applied to primary human BMSCs treated in osteogenic conditions for 11 days. As observed in the immortalised cell line, IL-1β supplementation (100 pg/ml) in primary cells led to a significant increase in mineral deposition (2.91 ± 0.15 fold change compared to OM), while IL-8 (2 ng/ml) and IL-10 (100 pg/ml) did not produce any notable changes (Figure 4).

### Dose-Response of IL-1β on Osteogenic Differentiation

**FIGURE 5:**
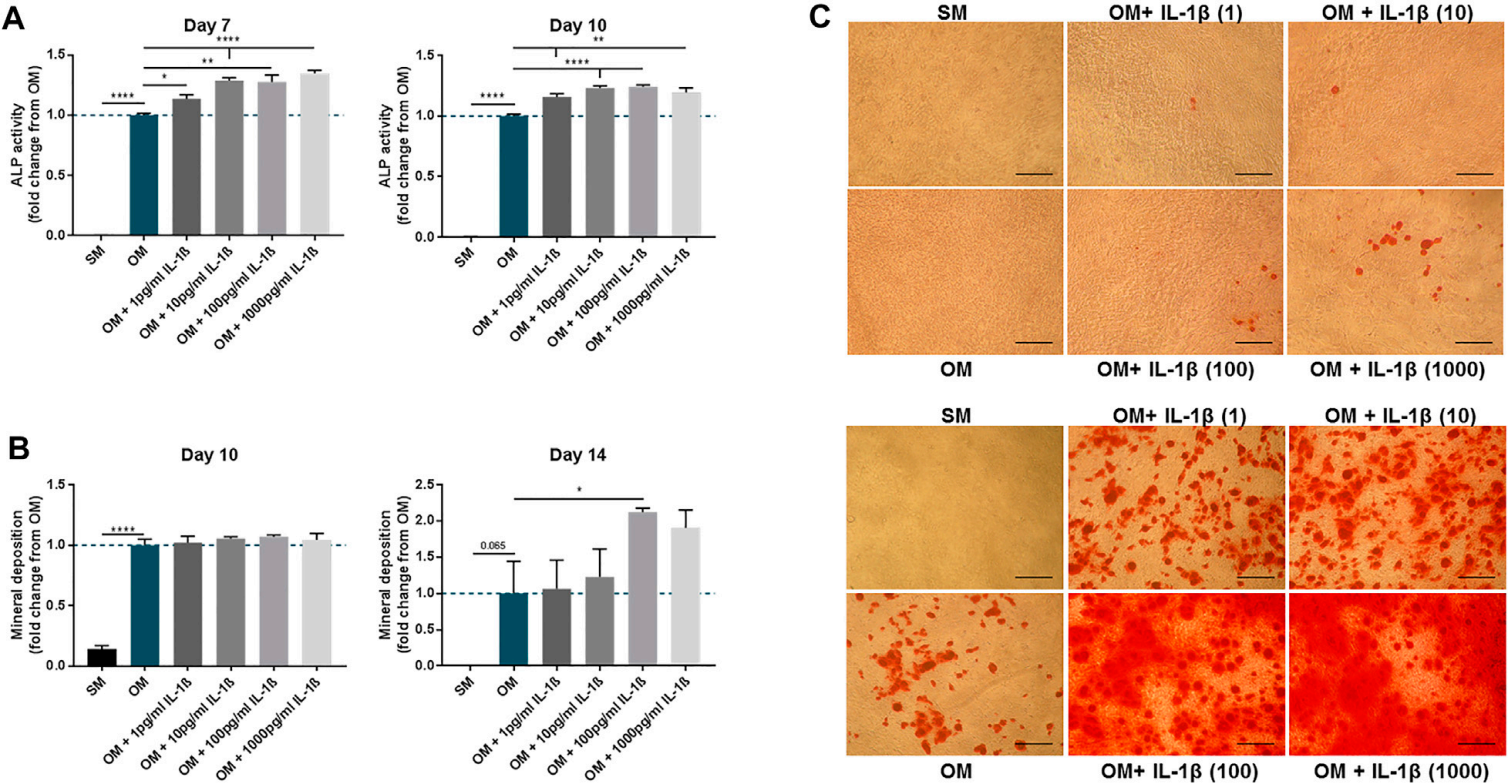
Dose-response of IL-1β (1–1,000 pg/ml) on MSC cultures undergoing osteogenic treatment. **(A)** ALP activity measured at day 7 and 10. **(B)** Mineral deposition measured at day 10 and 14. **(C)** Representative images of Alizarin Red staining at day 10 (upper panel) and 14 (lower panel). Scale bar = 150 μm. Data shown as fold change compared to the OM control condition. Error bars represent SEM. *n* = 4, *p < 0.05, **p < 0.01, ***p < 0.001, ****p < 0.0001.

The effect of IL-1β on MSC osteogenic response was further refined using doses ranging from 1 pg/ml to 1,000 pg/ml (Figure 5). An increase in ALP activity in response to IL-1β was observed at day 7 and day 10 for all doses, although beyond 10 pg/ml the response was largely similar (Day 7: 1 pg/ml 1.14 ± 0.04, 10 pg/ml 1.29 ± 0.02, 100 pg/ml 1.28 ± 0.06, 1,000 pg/ml 1.35 ± 0.03 fold change compared to OM; Day 10: 1 pg/ml 1.16 ± 0.03, 10 pg/ml 1.23 ± 0.02, 100 pg/ml 1.24 ± 0.02, 1,000 pg/ml 1.19 ± 0.04 fold change compared to OM). Analysis of mineral deposition at day 14 showed a positive response (Figure 5C), with a significant increase in OM conditions supplemented with IL-1β at 100 pg/ml (2.12 ± 0.06 fold change compared to OM) (Figure 5B). Interestingly, while mineral deposition did not show significant differences with any of the IL-1β doses at day 10 (Figure 5B), brightfield microscopy observation revealed that mineral deposition had begun in discrete areas of cultures exposed to IL-1β (Figure 5C).

### Effects of IL-1β Supplementation on MSC Gene Expression

**FIGURE 6:**
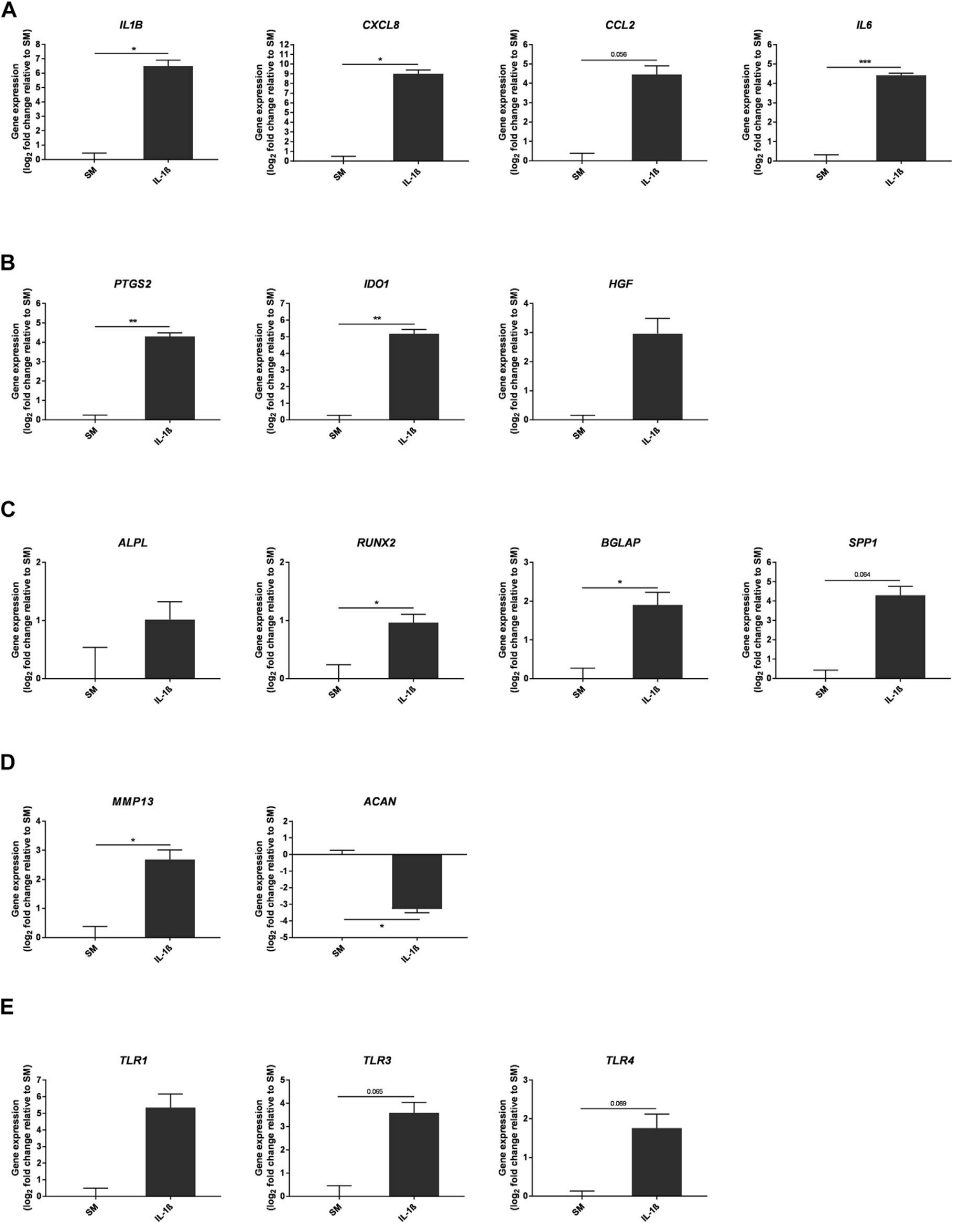
Gene expression changes in response to IL-1β (100 pg/ml) added to MSC cultures in SM over 10 days. Gene expression of pro-inflammatory **(A)** and immunomodulatory **(B)** mediators, osteogenic **(C)** and chondrogenic **(D)** factors, and toll-like receptors **(E)**. Data shown as log_2_ fold change compared to SM control condition. Error bars represent SEM. *n* = 3, *p < 0.05, **p < 0.01, ***p < 0.001.

To further characterise the response of MSCs to IL-1β, gene expression was assessed after 10 days of treatment under SM conditions *in vitro*, focusing on genes linked to inflammation and differentiation (Figure 6). Expression of pro-inflammatory mediators was increased with IL-1β treatment (*IL1B* 90.53 ± 29.07, *CXCL8* 518.4 ± 158.3, *CCL2* 21.97 ± 7.85, *IL6* 21.47 ± 1.62 fold change compared to SM), alongside expression of immunomodulatory genes (*PTGS2* 19.74 ± 2.59, *IDO1* 36.24 ± 7.28, *HGF* 7.8 ± 3.44 fold change compared to SM). Whilst IL-1β supplementation upregulated the expression of *ALPL*, *RUNX2*, *BGLAP* and *SPP1*, which are associated with osteogenic differentiation (*ALPL* 2.02 ± 0.47, *RUNX2* 1.95 ± 0.2, *BGLAP* 3.75 ± 0.94, *SPP1* 19.69 ± 7.37 fold change compared to SM), expression of the chondrogenic marker *ACAN* was decreased (0.1 ± 0.01 fold change compared to SM) and that of the cartilage degrading enzyme *MMP13* was increased (6.45 ± 1.62 fold change compared to SM). Furthermore, expression of toll-like receptor genes, including *TLR1*, *TLR3* and *TLR4*, which are important for the secretion of inflammatory mediators (DelaRosa et al., 2012; Sangiorgi and Panepucci, 2016; Shirjang et al., 2017; Barreto et al., 2020), were all increased (*TLR1* 40.72 ± 31.05, *TLR3* 12.08 ± 4.38, *TLR4* 3.38 ± 0.96 fold change compared to SM).

### Effects of IL-1β Supplementation on Mesenchymal Stem Cell Gene Expression During Chondrogenic Differentiation

**FIGURE 7:**
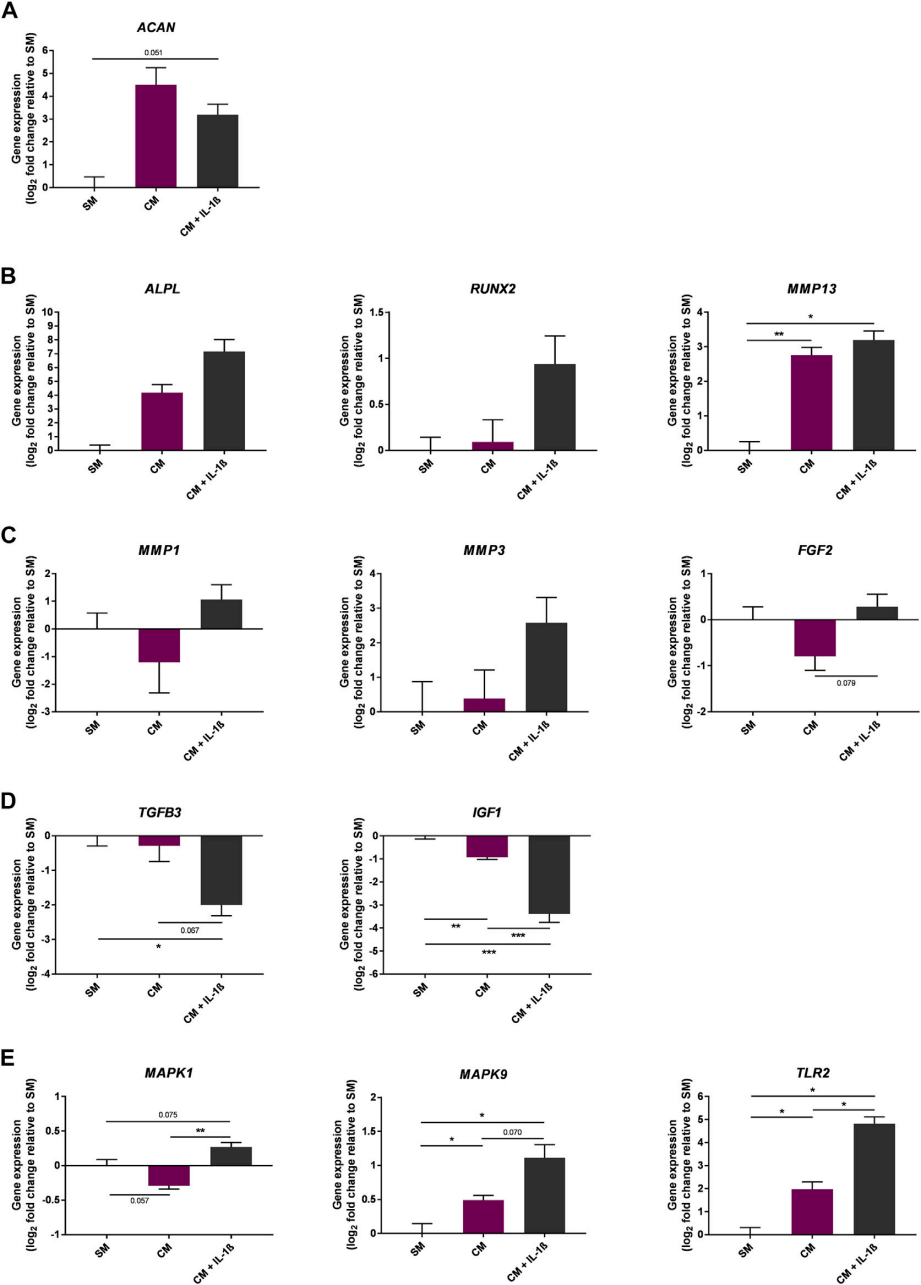
Gene expression changes in response to IL-1β (100 pg/ml) added to MSC cultures in CM over 10 days. Gene expression of chondrogenic **(A)** and chondrogenic hypertrophy **(B)** markers, catabolic **(C)** and anabolic **(D)** markers, and MAPK enzymes and toll-like receptors **(E)**. Data shown as log_2_ fold change compared to SM control condition. Error bars represent SEM. *n* = 3, *p < 0.05, **p < 0.01, ***p < 0.001.

The effect of IL-1β supplementation (100 pg/ml) on gene expression was also measured during chondrogenic differentiation of MSCs in CM medium (Figure 7). As expected, the expression of *ACAN* was increased at day 10 of the chondrogenic differentiation protocol (22.6 ± 15.22 fold change compared to SM); however the presence of IL-1β negatively impacted *ACAN* expression (9.12 ± 3.4 fold change compared to SM). In addition, expression of catabolic genes including cartilage degrading enzymes such as *MMP1*, *MMP3*, and the growth factor *FGF2* were increased upon supplementation with IL-1β (*MMP1* 2.09 ± 0.95, *MMP3* 5.98 ± 3.96, *FGF2* 1.22 ± 0.25 fold change compared to SM). In contrast, genes encoding anabolic growth factors such as *TGFB3* and *IGF1* showed a marked decrease in expression upon IL-1β supplementation (*TGFB3* 0.25 ± 0.05, *IGF1* 0.1 ± 0.02 fold change compared to SM). Of note, the expression of genes encoding for chondrocyte hypertrophy markers such as *RUNX2*, *ALPL* and *MMP13* showed an upward trend following IL-1β supplementation (*RUNX2* 1.92 ± 0.45, *ALPL* 144.4 ± 115.9, *MMP13* 9.18 ± 1.84 fold change compared to SM), alongside increased expression of *MAPK1*, *MAPK9* and *TLR2*, which are associated with increased expression of matrix metalloproteinases (MMPs) and pro-inflammatory mediators (*MAPK1* 1.2 ± 0.06, *MAPK9* 2.17 ± 0.3, *TLR2* 28.18 ± 6.44 fold change compared to SM) (Wang et al., 2011; DelaRosa et al., 2012; Mariani et al., 2014; Sangiorgi and Panepucci, 2016; Barreto et al., 2020).

### Effects of IL-1β Supplementation on Mesenchymal Stem Cell Gene Expression During Osteogenic Differentiation

**FIGURE 8:**
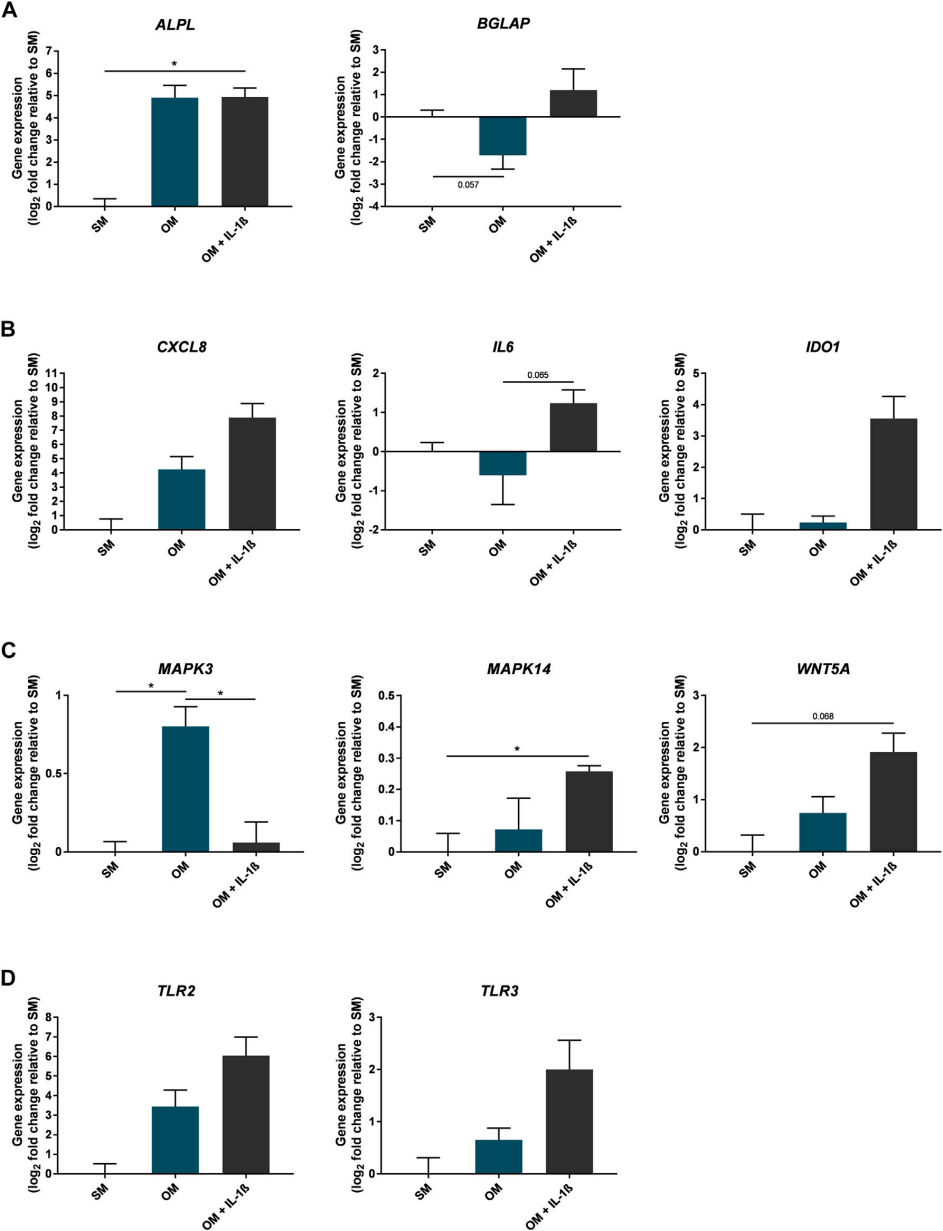
Gene expression changes in response to IL-1β (100 pg/ml) added to MSC cultures in osteogenic medium over 10 days. Gene expression of osteogenic **(A)**, inflammatory mediators **(B)**, MAPK enzymes and Wnt signalling factors **(C)**, and toll-like receptors **(D)**. Data shown as log_2_ fold change compared to SM control condition. Error bars represent SEM. *n* = 3, *p < 0.05.

To further determine the effect of IL-1β on MSC osteogenesis *in vitro*, gene expression was assessed in cells at day 10 of osteogenic differentiation (Figure 8). The early osteogenic differentiation marker *ALPL* increased in response to osteogenic conditions (29.96 ± 13.85 fold change compared to SM), including with supplementation of IL-1β (100 pg/ml) (30.56 ± 9.72 fold change compared to SM). In contrast, expression of *BGLAP*, which is important for the late mineral deposition stage, was increased following supplementation of IL-1β (2.31 ± 2.14 fold change compared to SM), as was the expression of genes encoding inflammatory mediators (*CXCL8* 236.7 ± 229.7, *IL6* 2.37 ± 0.62, *IDO1* 11.75 ± 7.45 fold change compared to SM). Expression of genes involved in the regulation of bone remodelling and the production of inflammatory mediators (*MAPK3*, *MAPK14*, *TLR2*, *TLR3*, *WNT5A*) (Jaiswal et al., 2000; Sonomoto et al., 2012; Yamashita et al., 2012; Artigas et al., 2014; Qi et al., 2014; Muthukuru and Darveau, 2015; Kobayashi et al., 2016; Sangiorgi and Panepucci, 2016; Shirjang et al., 2017; Amarasekara et al., 2021) were also altered in the presence of IL-1β (*MAPK3* 1.04 ± 0.1, *MAPK14* 1.2 ± 0.02, *TLR2* 66.38 ± 61.57, *TLR3* 4 ± 1.9, *WNT5A* 3.76 ± 1.09 fold change compared to SM).

## Discussion

The intrinsic regenerative properties of MSCs may allow these cells to repair cartilage, as well as dampen inflammation in the joint due to their recognised local immunosuppressant effects (Gao et al., 2016; Wang et al., 2018; Harrell et al., 2019; Song et al., 2020; Zhao et al., 2020; Hwang et al., 2021; Zhu et al., 2021; Xiang et al., 2022). However, MSC transplantation attempts in OA clinical trials have thus far produced limited evidence of cartilage regeneration, despite reported improvements in pain and physical function (Iijima et al., 2018; Wang et al., 2020; Zhu et al., 2021). This might be due to the hostile local microenvironment encountered in the OA joint, where the presence of inflammatory mediators such as interleukins can contribute to disease progression (Kapoor et al., 2011; Wojdasiewicz et al., 2014; Molnar et al., 2021). Although the effect of inflammatory mediators on transplanted cells remains unclear, their role in altering the phenotype and differentiation potential of endogenous MSC populations in the OA joint has been suggested (Murphy et al., 2002; Coleman et al., 2010; Jayasuriya et al., 2018), thereby limiting the therapeutic potential of exogenous MSCs to mitigate disease progression. On that basis, analysing how specific mediators present in the affected joint impact on MSC function is required to inform novel cell-based therapies for OA. The present study investigated how exposure to IL-1β (Westacott et al., 1990), IL-8 (Kaneko et al., 2000) and IL-10 (Helmark et al., 2010), which are upregulated in the OA joint, affected MSC phenotype and potential by measuring parameters of cell proliferation, differentiation and gene expression. Results demonstrated that while standard concentrations of IL-8 and IL-10 did not affect the proliferation or differentiation capacity of MSCs, the addition of IL-1β significantly affected the differentiation of MSCs *in vitro*.

### IL-1β Effect in Chondrogenic Conditions

Exposure to IL-1β was observed to impede the chondrogenic response of MSCs *in vitro*, with a notable reduction in sGAG production. This was consistent with decreased *ACAN* expression compared to untreated controls, supporting the association between reduced GAG synthesis and lower proteoglycan gene expression (Pfander et al., 2004). In parallel to this negative effect on chondrogenesis, IL-1β is also thought to exert some negative effects in the joint through degradation of extracellular matrix (ECM) components, a key feature of OA cartilage breakdown (Martel-Pelletier, 2004; Goldring and Marcu, 2009). This is supported by the increased expression of the growth factor *FGF2*, reported to have catabolic effects in human articular chondrocytes (Li et al., 2012), as well as cartilage degrading enzymes including *MMP1*, *MMP3* and *MMP13*, upon IL-1β treatment. In the OA joint, secretion of these proteases indicates the progression of chondrocytes towards a state of hypertrophy, which subsequently stimulates endochondral ossification processes linked to the development of painful osteophytes (Van der Kraan and Van den Berg, 2012). Although *in vitro* systems of chondrogenic differentiation are intrinsically limited by the spontaneous progression of MSCs towards the hypertrophic stage (Zhang et al., 2010; Chen et al., 2015), data presented here show that exposure to IL-1β-rich conditions upregulated chondrocyte hypertrophy markers such as *RUNX2*, *ALPL* and *MMP13* in MSCs. Gene expression analysis also highlighted changes in *MAPK1*, *MAPK9* and *TLR2* expression following IL-1β exposure, thereby indicating alterations to signalling pathways known to augment expression of MMPs and pro-inflammatory mediators (Wang et al., 2011; Mariani et al., 2014; Sangiorgi and Panepucci, 2016; Barreto et al., 2020). In parallel, IL-1β treatment reduced the expression of *TGFB3* and *IGF1*, known to promote the synthesis of ECM proteins (Bohme et al., 1992; Tang et al., 2009) and contribute to cartilage maintenance in the healthy joint. The consequences of such changes should now be further explored, considering the ensuing risk of transplanted MSCs contributing to cartilage degradation when exposed to an OA environment.

### IL-1β Effect in Osteogenic Conditions

In addition to cartilage degradation, abnormal bone remodelling is also a key feature of OA pathogenesis (Donell, 2019; Hu et al., 2020), and is linked to increased pain in this condition (Nwosu et al., 2016; Hu et al., 2021). Here, IL-1β was observed to significantly enhance the osteogenic response of MSC cultures, with a notable induction of ALP activity and mineral deposition. These findings were corroborated by the upregulation of *BGLAP* expression, a gene involved in the later mineralisation stage of osteogenesis (Birmingham et al., 2012). IL-1β addition to osteogenic medium also increased *WNT5A* expression. This is in line with previous observations of a pro-osteogenic effect of IL-1β on human MSCs, reported to be mediated primarily through the Wnt-5a/receptor tyrosine kinase-like orphan receptor 2 pathway (Sonomoto et al., 2012). Both canonical and non-canonical Wnt signalling play an important role in bone formation (Kim et al., 2015; Kobayashi et al., 2016; Maeda et al., 2019). However, in the OA joint, osteoblasts display reduced canonical Wnt/β-catenin signalling and increased non-canonical Wnt signalling via Wnt5a (Martineau et al., 2017), a ligand also linked to enhanced osteoclastogenesis and the production of inflammatory mediators (Yamashita et al., 2012; Roberts et al., 2020). Similarly, *TLR2*, *TLR3* and *MAPK14* showed increased expression upon IL-1β exposure, and are associated with increased osteogenesis (Artigas et al., 2014; Qi et al., 2014; Muthukuru and Darveau, 2015), whilst simultaneously promoting the production of pro-inflammatory mediators linked to osteoclastogenesis (Patil et al., 2004; Sangiorgi and Panepucci, 2016; Amarasekara et al., 2021). These include IL-8 (CXCL8) and IL-6, which both showed increased gene expression upon IL-1β exposure in this study. The observed decrease in *MAPK3* expression upon IL-1β exposure compared to OM could be linked to the accelerated time-course of osteogenic response observed in these cells, as expression of this signalling molecule is important during early differentiation stages before rapidly returning to baseline (Jaiswal et al., 2000; Li et al., 2016). Taken together, this could implicate osteogenic MSCs exposed to IL-1β as a contributing factor in the increased rate of bone resorption and remodelling observed in OA.

### IL-1β Effect in Undifferentiated Conditions and Relevance for Immune Modulation

This preferential drive towards osteogenesis was also observed when IL-1β was applied to MSCs in their undifferentiated state. IL-1β exposure under standard culture conditions led to increased expression of genes involved in bone remodelling, including *ALPL*, *RUNX2*, *BGLAP* and *SPP1*, whilst genes involved in cartilage anabolism (*ACAN*) and catabolism (*MMP13*) were decreased or increased, respectively. It can thus be hypothesised that IL-1β primes MSCs towards the osteogenic lineage, which could have a detrimental outcome for MSCs transplanted into the OA joint for cartilage regeneration. Furthermore, since IL-1β treatment of undifferentiated MSCs also increased expression of genes encoding pro-inflammatory mediators such as IL-1β, IL-6, IL-8 and MCP-1, it can be hypothesised that MSCs exposed to an IL-1β-rich environment could secrete increased levels of mediators that contribute to inflammatory joint disease (Kapoor et al., 2011; Wojdasiewicz et al., 2014; Molnar et al., 2021). The upregulated expression of *PTGS2* (which encodes COX-2) in IL-1β-treated MSCs supports this view since both IL-6 and COX-2 are linked to pain in OA (Laine et al., 2008; Lin et al., 2017). Upregulation of *IDO1* and *HGF*, also observed here in MSCs exposed to IL-1β, has previously been correlated to OA disease. In particular, increased HGF has been observed in OA osteoblasts (Abed et al., 2015), and IDO1 is overexpressed in the synovial fluid of OA patients (Alahdal et al., 2020), supporting the hypothesis that the response to IL-1β observed in MSC cultures aligns with pathological features seen in OA.

Based on the present observations, it will be useful to extend the scope of the analysis and, in particular, to assess differentiation in MSCs exposed to combinations of different mediators which are found in the OA joint. The present report analysed the effect of single factors over a defined time-scale in culture, which could be extended in subsequent studies to monitor the longer term effect on MSC response, adding further differentiation markers detectable through protein expression assays to refine the phenotypic characterisation.

### Clinical Relevance

This study provides further evidence of the value of MSCs as a preclinical model, to evaluate specific pathophysiological factors and assess their impact on cell populations of therapeutic interest. The results presented now call for further investigations with increased experimental complexity, through the combined application of multiple inflammatory mediators present in the OA joint, to assess possible synergistic or antagonistic effects.

In conclusion, these results underline that IL-1β, a pro-inflammatory mediator associated with OA pathology, directly impacts the differentiation potential of MSCs *in vitro*, through a reduction in their chondrogenic capacity combined with a notable induction of osteogenesis. This suggests that the exposure of transplanted MSCs to this interleukin in the OA joint could promote subchondral bone remodelling rather than cartilage regeneration. Gene expression analysis highlighted changes in TLR, MAPK and Wnt signalling pathways and revealed increased expression of genes encoding pro-inflammatory mediators and cartilage-degrading enzymes, further highlighting the likely detrimental impact of the local inflammatory OA environment on transplanted MSCs. Given the widespread presence of IL-1β in the OA joint, it could indeed be hypothesised that the OA microenvironment may limit the therapeutic potential of MSCs, and favour their participation in the progression of OA pathology. These observations call for further investigation into the effects of the OA microenvironment on candidate therapeutic cell populations, in order to evaluate the expected benefit of these cell-based approaches.

## Data Availability

The original contributions presented in the study are included in the article, further relevant inquiries can be directed to the corresponding author.
